# *In-vitro* Evaluation of toothpaste containing enzymes and proteins on inhibiting plaque re-growth of the children with high caries experience

**DOI:** 10.4317/jced.57326

**Published:** 2021-01-01

**Authors:** Bhojraj Nandlal, Neelankavil-Kochouseph Anoop, Veeramani Ragavee, Lobo Vanessa

**Affiliations:** 1 Professor and Head, Department of Pediatric and Preventive Dentistry, JSS Dental College and Hospital, JSS Academy of Higher Education & Research, Mysore, Karnataka, India; 2 Department of Pediatric and Preventive Dentistry, JSS Dental College and Hospital, JSS Academy of Higher Education& Research, Mysore, Karnataka, India

## Abstract

**Background:**

Dental caries belong to a disease which has a complex etiology. Individual preventive measures are not adequate enough to control the disease especially in children with high caries experience The aim of the study was to evaluate the efficacy of tooth paste containing enzymes and proteins in inhibiting plaque re-growth when compared to fluoride tooth paste in children with high caries experience.

**Material and Methods:**

A total of 15 plaque samples are collected from 15 subjects in the age group of 5-7 years with High caries experience. The participants were asked to fast overnight for 12 h. On the day of the study, the participants were asked to report without brushing. Pre brushing pooled plaque was collected from buccal and palatal/lingual surfaces of maxillary and mandibular teeth and transferred to chilled Eppendorf tubes. Samples were divided into two groups; according to the test toothpaste used; Zendium toothpaste and Colgate cavity protection along with controls. Inhibition of plaque re-growth is assessed by comparing optical density using a spectrophotometer at intervals between 0-8 hours.

**Results:**

Results demonstrated that toothpaste containing enzymes and proteins can significantly inhibit plaque growth when compared to normal toothpaste (*p*<0.05) in children with high caries experience.

**Conclusions:**

Toothpaste containing enzymes and proteins can augment natural salivary defenses and control plaque re-growth which plays a major role in dental caries formation.

** Key words:**Enzymes, proteins, toothpaste, children, caries, dental plaque.

## Introduction

The human body’s resident micro biota is not only essential for life but also plays a crucial role in both the protection from, and development of, various diseased states (1). Three quarters of all microbial infections found in humans are associated with microbial biofilm (2) for instance oral diseases, dental caries and periodontal diseases caused by oral biofilm known as dental plaque. Dental caries is a health problem in a majority of developed countries (mainly due to cariogenic diet) and, as shown by a report of the World Health Organization, it affects people of all ages, i.e. 60 - 90% of school-age children and most adults (3).

Dental caries belongs to a group of diseases that are considered “complex” or “multifactorial,” with no single causation pathway, and therefore, are not amenable to simplistic preventive solutions such as the elimination of “one type of organism” or merely enhancing “tooth resistance” (4). The control and prevention of any disease should preferably focus on the etiological factors involved. For dental caries, this would be the periodic disorganization of the oral plaque biofilm by mechanical oral hygiene, along with dietary modification to reduce exposure to fermentable carbohydrates (5). Unfortunately, individual oral hygiene measures have only a limited impact in caries prevention. While minimizing the etiological factors contributing to the disease is critical, additional preventive measures commensurate with individual risk status may still be required in many segments of the population especially with high caries risk.

Saliva plays an important role in preventing dysbiosis and maintaining health in the oral cavity (6). Salivary components, particularly antimicrobial factors such as enzymes and proteins, exert significant selective pressures on the micro biota, helping to provide protection against pathogenic organisms and helping to shape and control the resident community. One of the main defense mechanisms of saliva is the lactoperoxidase system (LPO system) (7). In addition to the LPO system, other salivary components including lysozyme and lactoferrin are critical to the mouth’s natural defenses against bacteria (8).

To boost the role of natural salivary defenses in controlling the oral microbial community, oral hygiene products including toothpastes have been developed that contain enzymes and proteins.

In the current study, commercially available toothpaste containing enzymes and proteins, Zendium™ has been tested. Zendium contains a three enzyme system (amyloglucosidase, glucose oxidase and lactoperoxidase), designed to promote the generation of hydrogen peroxide and hypothiocyanite, as well as three further protein components (lysozyme, lactoferrin and immunoglobulin) designed to provide additional antimicrobial benefits compared to control fluoride toothpaste without the enzymes and proteins.

Most of the previous studies were done using microbial plating method or else chair‑side method. These methods have been used for many decades and constitute the conventional method of antimicrobial analysis but are not very accurate or reproducible. The Plaque Glycolysis and Re-growth Method (PGRM) was used for the evaluation of the antimicrobial effects on plaque metabolism (9).

The aim of the study was to evaluate efficacy of Fluoride tooth paste containing 

Enzymes and Proteins in inhibiting plaque re-growth when compared to fluoride tooth paste also to evaluate plaque re-growth by comparing Optical Density using a spectrophotometer at intervals between 0-8 hours.

## Material and Methods

The Study was approved by the Institutional ethical committee. The entire procedure was done at SIG Dental Cariology lab of Department of Pedodontics and Preventive Dentistry. The sample size was calculated using G Power software. It was calculated to be 15 assuming 80% power, an alpha error of 0.05 and effect size ranging between 0.8 and 1. A total of 15 plaque samples are collected from 15 subjects in the age group of 5-6 years with High caries experience (dmft > 5) and without unerupted tooth and all teeth present for the age. Dental examinations were conducted after teeth were air-dried, under artificial light and with the aid of a dental mirror and explorer. The number of teeth was recorded for each patient. All erupted teeth were evaluated according to the criteria recommended by the World Health Organization (WHO) using the dft and DMFT index. Children who have under gone previous dental treatment, teeth with acute conditions, children with systemic diseases, children with learning disabilities and those under antibiotic therapy either at the time of the study or up to 3 months before the start of the study were excluded. Parents were informed about methodology of the study and written consent was obtained from the participant and parent.

The participants were asked to fast overnight for 12 h before the start of study. On the day of the study, the participants were asked to report without brushing at 8 am. Pre brushing pooled plaque was collected from buccal and palatal/lingual tooth surfaces of maxillary and mandibular teeth and transferred to autoclaved Eppendorf tubes which were placed in the mini cooler at −20°C.

The stock solutions of 6% and 0.03% Trypsin Soy Broth (TSB), distilled water and 40% sucrose solutions were prepared the day before the study and autoclaved. Plaque dispersion was done in 1.75 ml of 0.03% TSB at ph 7.1 by vortexing for 3 min and biomass was standardized to 0.2 OD by adding the required amount of 0.03% TSB.

The test toothpaste, Zendium contains three enzyme and three protein components, along with Sodium fluoride (1450ppm) and the control toothpaste, Colgate cavity protection contain Sodium monoflurophosphate 0.76% (0.15%w/v fluoride ions) 1000ppm. 20 mg/ml stock concentration of toothpaste was prepared and it was diluted in 1:10 ratio for the final working concentration of 2mg/ml.

For the purpose of the study three groups were formulated i.e. the positive control, F+Enzyme + Protein Toothpaste (fluoride+ enzymes + protein toothpaste), F toothpaste (Fluoride toothpaste). The prepared master mix of each group was aliquoted in 1050µl for each tube of 0hrs, 2hrs, 4hrs, 6hrs, and 8hrs and marked respectively.

The triplicates of each sample were taken to give total of 45 samples in each of the three group so as to give total of 135 samples. The mean of the OD of triplicates are taken at each time point.

For statistical analysis, Test of significance was done using recent version of SPSS software 23.

-Procedure of Quantification of Bacterial Load by Spectrophotometer

The 0 hours samples were individually pipetted out and transferred to optical cuvette, taken measuring optical density at 600 nm using a spectrophotometer and to measure the ph of solution using a ph meter. All the samples of other time periods were kept in incubator shaker of 300 rpm. Similarly, OD and pH were noted for each sample at 2hrs, 4hrs, 6hrs, and 8hrs, respectively, using a spectrophotometer.

## Results

The baseline Optical Density of all samples at all the time periods were standardized at 0.2 OD and the change in the OD in different time point are tabulated (Table 1) The mean OD of F+Enzyme + Protein Toothpaste, positive control and the F toothpaste at 6 hrs are 0.864±0.183, 1.126±0.151 and 1.187±0.164 respectively and at 8 hrs are 1.037±0.188, 1.233±0.203 and 1.191±0.233 respectively. It was seen that the mean OD of F+Enzyme + Protein Toothpaste is lower than the positive control and the F toothpaste. Also, the same pattern is seen in the mean ph values (Table 2). The F+Enzyme + Protein Toothpaste group was less acidic than F toothpaste and the positive control.

**Table 1:**
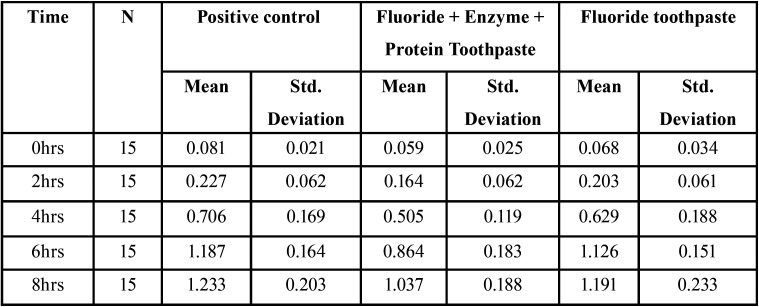
Change in OD from base line to 2 hrs, 4hrs, 6 hrs and 8hrs.

**Table 2:**
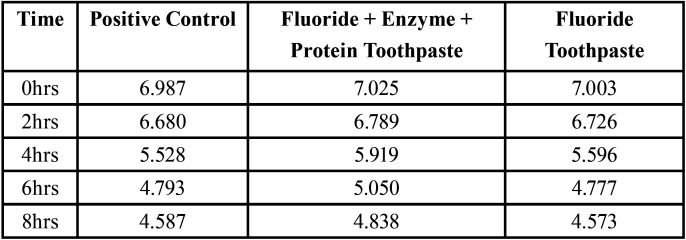
Assessment Of Plaque Re-growth based on change in pH.

The study indicates that there was a significant difference between F +Enzyme + Protein toothpaste and F toothpaste at 4 hrs and 6 hr at 5 % level and no significant difference was observed at 0hrs, 2hrs and 8hrs (Table 3).

**Table 3:**
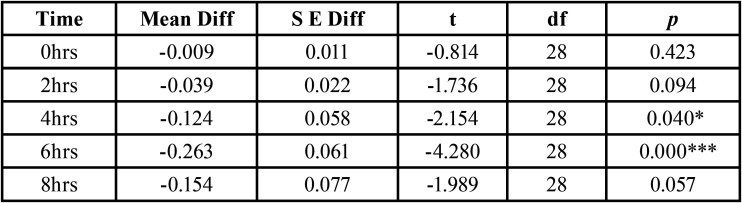
T Test between OD of Fluoride + Enzyme + Protein Toothpaste and Fluoride Toothpaste.

Table 4 shows the percentage inhibition (Percentage inhibition = (OD of test – OD of control )/(OD of Control) x 100) of bacterial growth between the F+Enzyme+Protein Toothpaste and F toothpaste, and the control. The percentage inhibition of bacterial growth in F+Enzyme+Protein Toothpaste with positive control at 2hrs, 4hrs, 8hrs are 27.75,28.17,27.21and 15.89 respectively. The percentage inhibition of bacterial growth in F Toothpaste with positive control at 2hrs, 4hrs, 8hrs are 10.57, 10.90, 05.13 and 03.40 respectively. Highest difference in percentage inhibition between the two toothpastes was seen at 6hrs.

**Table 4:**
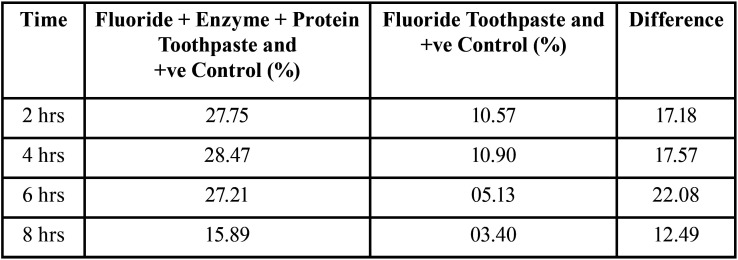
Percentage Inhibition of Fluoride + Enzyme + Protein Toothpaste And Fluoride Toothpaste With Control.

## Discussion

As described by Kilian *et al.*, humans have co-evolved with microorganisms and have a symbiotic or mutualistic relationship with their resident micro biome which, for the most part, remains homeostatic(10). The symbiotic relationship can break down, for example, through poor oral health regimes, resulting in dysbiosis and plaque-related diseases (11). Lactoperoxidase system, one of the defense mechanism of saliva is activated in part by hydrogen peroxide which oxidizes thiocyanate to hypothiocyanite, Hydrogen peroxide itself possesses antimicrobial activity in the oral cavity which is produced by both the human host and members of the oral microbiome (12) Tenovuo *et al.* have demonstrated the antibacterial effect of salivary peroxidases on a cariogenic strain of *Streptococcus mutans* (13). Hypothiocyanite produced by the LPO system has antibacterial effects on both cariogenic bacteria and black pigmented anaerobic bacteria associated with periodontal disease. Lysozyme is an antibacterial protein found in a variety of mucosal fluids. Quantitatively, it is the most important salivary component with antibacterial properties, due to its ability to break glycosidic linkages in peptidoglycans. Lactoferrin has been shown to permeabilise the outer membrane of Gram-negative bacteria making them susceptible to penetration by lysozyme. The effect is most pronounced against Gram-positive bacteria due to the thick peptidoglycan layer in the cell wall (6). In addition to its main mode of action lactoferrin acts as an iron-binding protein which reduces the concentration of iron available as a co-factor for bacterial enzymes and in turn retards bacterial growth. As well as bacteriostatic properties, lactoferrin is known to have bactericidal properties in its own right resulting from direct interaction between the protein and bacteria.(14) Lenander and Loimaranta stated that the main oral innate defense factors are the peroxidase systems, lysozyme, lactoferrin, and histatins (15).

According to study done by S. E. Adams *et al.* in 2017 on adult population, Zendium, toothpaste containing enzymes and proteins helps In augmenting the natural salivary defenses. The present study which is the first of its kind, used tooth paste containing enzymes and proteins in certain segments of population i.e. the children with high caries experience where conventionally followed techniques are not adequate enough to control the microbial population (16).

Furthermore, the methodology followed, though not a recent concept was found to be rarely used. The PGRM methodology was first introduced in 1995 by White *et al.* This methodology primarily relies on the experimental observation that natural fasted dental plaque, sampled from different quadrants of the dentition, exhibits similar metabolic and regrowth properties when suspended at equal “biomass” in standardized media. This method is more accurate and less cumbersome than the traditional plate culturing method of bacterial growth (9).

During the course of the study, six groups were formed where the negative control is kept so as to rule out any contamination throughout the study, positive control helps in determining the plaque regrowth without any antimicrobial agents. Paste control 1 and paste control 2 are control groups for Zendium and Colgate cavity protection respectively in which OD values remained constant throughout the study.

The pH value of the samples was taken along with the OD so as to correlate the growth of the microorganisms in the plaque. Higher the re-growth lesser the pH due to the acid produced during the bacterial metabolism. As we see in the results, the pH value was more towards acidic in the control followed by F toothpaste and then in F+Enzyme+Protein Toothpaste. This shows lesser bacterial growth in F+Enzyme +Protein Toothpaste when compared to others.

Statistical analysis showed that there is significant difference in the OD between F+Enzyme + Protein Toothpaste and F toothpaste at 4 and 6 hrs and a similar trend can be seen in the difference in percentage inhibition between F+Enzyme+ Protein Toothpaste and F toothpaste where the highest is seen at 6hrs. F+enzymes+proteins toothpaste shows significant difference when compared to positive control in inhibition of plaque re-growth at all time periods in high caries group. F toothpaste, though the means were lower than the positive control didn’t show any significant difference when compared to positive control which shows that fluoride containing tooth paste is less effective in controlling plaque regrowth in high caries experience children where the bacterial load is too high.

This being an *in vitro* study, the buffering capacity of the saliva in the normal mouth on exposure to the sucrose challenge could not be taken into account. Further *in vivo* studies are required to study the exact process occurring clinically.

The very high concentration of sucrose solution (40%) used in the study can be justified as it permits us to reduce the duration of the study at the same time allowing us to extrapolate the results to the normal sugar consumption levels. On an average, a person consumes 4 g (2 teaspoons) of sugar with the regular beverage. Thus, in this study, 10 times the normal concentration of sugar exposure at any given point of time was added.

The results of the present study suggest that the F+enzymes+protein toothpaste is effective in reducing the plaque growth of the children with high caries experience up to 6 hrs when immediately exposed to 40% sucrose load.

From this study, we can conclude that Zendium which is a fluoridated toothpaste containing enzymes and proteins is more effective in inhibiting plaque re-growth when compared to fluoride toothpaste and can augment natural salivary defenses to control dental caries which plays a major role in high caries experience group.

However, one must bear in mind that the effective ness of the toothpastes may be may be quite different in clinical scenario and further research as *in vivo* studies are required to substantiate these results.
